# Supplementary material for: Unveiling the Plasmodium inositol (pyro)phosphate pathway: Highlighting inositol polyphosphate multikinase as a novel therapeutic target for malaria
Source: PLoS One. 2025 Dec 10;20(12):e0338411. doi: 10.1371/journal.pone.0338411 (PMC12694804; doi:10.1371/journal.pone.0338411)
Supplement: S1 File — Sequences from P. falciparum (Q8I3W0), P. malariae (A0A1D3RII6), P. vivax (A0A564ZW73), P. ovale (A0A1D3U8P3) and P. knowlesi (B3L6B8) were aligned with Clustal Omega and viewed in Jalview. The sequences in the grey frame highlight the identified signature motifs QxxxDxKxG, SLL and IDF. Invariant residues are shaded blue. S1 Table. List of IPK proteins used for structural analysis and their annotations. S2 Fig. Modelling and structure assessment of P. falciparum IPMK1. (A & C) Local distance difference test (LDDT) score per position predicted for the five P. falciparum IPMK1_kinase domain models generated by Colabfold. The model predictions had high- and low-confidence regions, and the low-confidence areas (red, blue and orange frames) were intrinsically disordered regions. (B) PAE plot of the best model predicted, which was refined and analysed on ProSA-Web (E) and SAVES (D), showing that 94.5% was Ramachandran favoured. S3 Fig. 3D modelling of the PfIPMK kinase domain by SWISS-MODEL. (A) Template information. (B) Model assessment of the predicted P. falciparum IPMK2 domain showing a Ramachandra plot of the initial model structure and that of the refined model, which has 94.4% of its residues in the favoured regions (D). (C) ProSA-web output of the quality assessment of the refined model. S2 Table. Table of druggable pockets predicted by PockDrug for P. falciparum IPMK2 and their parameters. S3 Table. Summary of the predicted subcellular locations of the putative P. falciparum IPKs. S1 Sequence. Predicted amino acid sequence of P. falciparum 3D7 IPMK1. S4 Table. Peptides mapped to enzymes in the P. falciparum IPP. S1 document. Uncropped images. (ZIP) [file pone.0338411.s001.zip › Supp_Information/S4 Table.pdf]

S4 Table: Peptides mapped to enzymes in the *P. falciparum* IPP

Stage	IPMK	IP6K	VIP1	References
Blood stage schizonts	YES	YES	YES	[1]
Intraerythrocytic stage	YES	YES	YES	[2]
Merozoites	YES	YES	YES	[3]
Cytoplasmic and nuclear fraction from ring, trophozoites and schizonts	YES	ND	ND	[4]
Sexual and asexual life cycle	YES	ND	YES	[5]
Salivary gland sporozoites	YES	ND	YES	[6]
Sporozoites surface	ND	ND	YES	[7]
Gametocyte	ND	ND	YES	[8]
Stage IV gametocyte	YES	ND	ND	[9]
Stage V gametocyte	YES	YES	YES	[9]
Oocysts sporozoites	YES	ND	YES	[10]

ND- Not detected

Supporting References

1. Treeck M, Sanders JL, Elias JE, Boothroyd JC. The phosphoproteomes of *Plasmodium falciparum* and *Toxoplasma gondii* reveal unusual adaptations within and beyond the parasites' boundaries. Cell Host Microbe. 2011;10(4):410-419. doi: 10.1016/j.chom.2011.09.004. PMID: 22018241; PMCID: PMC3254672.

2. Pease BN, Huttlin EL, Jedrychowski MP, Talevich E, Harmon J, Dillman T, et al. Global analysis of protein expression and phosphorylation of three stages of *Plasmodium falciparum* intraerythrocytic development. J Proteome Res. 2013;12(9):4028-4045. doi: 10.1021/pr400394g. PMID: 23914800; PMCID: PMC5292867.

3. Lasonder E, Green JL, Grainger M, Langsley G, Holder AA. Extensive differential protein phosphorylation as intraerythrocytic *Plasmodium falciparum* schizonts develop into extracellular invasive merozoites. Proteomics. 2015;15(15):2716-2729. doi: 10.1002/pmic.201400508. PMID: 25886026.

4. Oehring SC, Woodcroft BJ, Moes S, Wetzel J, Dietz O, Pulfer A, et al. Organellar proteomics reveals hundreds of novel nuclear proteins in the malaria parasite *Plasmodium falciparum*. Genome Biol. 2012;13(11):R108. doi: 10.1186/gb-2012-13-11-r108. PMID: 23181666; PMCID: PMC4053738.

5. Florens L, Washburn MP, Raine JD, Anthony RM, Grainger M, Haynes JD, et al. A proteomic view of the *Plasmodium falciparum* life cycle. Nature. 2002;419(6906):520-526. doi: 10.1038/nature01107. PMID: 12368866.

6. Lindner SE, Swearingen KE, Harupa A, Vaughan AM, Sinnis P, Moritz RL, Kappe SH. Total and putative surface proteomics of malaria parasite salivary gland sporozoites. Mol Cell Proteomics. 2013;12(5):1127-1143. doi: 10.1074/mcp.m112.024505. PMID: 23325771; PMCID: PMC3650326.

7. Swearingen KE, Lindner SE, Shi L, Shears MJ, Harupa A, Hopp CS, et al. Interrogating the Plasmodium sporozoite surface: identification of surface-exposed proteins and demonstration of glycosylation on CSP and TRAP by mass spectrometry-based proteomics. PLoS Pathog. 2016;12(4):e1005606. doi: 10.1371/journal.ppat.1005606. PMID: 27128092; PMCID: PMC4851412.

8. Lasonder E, Janse CJ, van Gemert GJ, Mair GR, Vermunt AM, Douradinha BG, et al. Proteomic profiling of Plasmodium sporozoite maturation identifies new proteins essential for parasite development and infectivity. PLoS Pathog. 2008;4(10):e1000195. doi: 10.1371/journal.ppat.1000195. PMID: 18974882; PMCID: PMC2570797.

9. Silvestrini F, Lasonder E, Olivieri A, Camarda G, van Schaijk B, Sanchez M, et al. Protein export marks the early phase of gametocytogenesis of the human malaria parasite Plasmodium falciparum. Mol Cell Proteomics. 2010;9(7):1437-1448. doi: 10.1074/mcp.m900479-mcp200. PMID: 20332084; PMCID: PMC2938084.

10. Lindner SE, Swearingen KE, Shears MJ, Walker MP, Vrana EN, Hart KJ, et al. Transcriptomics and proteomics reveal two waves of translational repression during the maturation of malaria parasite sporozoites. Nat Commun. 2019;10(1):4964. doi: 10.1038/s41467-019-12936-6. PMID: 31673027; PMCID: PMC6823429
