# Supplementary figures and images for: Unveiling the Plasmodium inositol (pyro)phosphate pathway: Highlighting inositol polyphosphate multikinase as a novel therapeutic target for malaria
Source: PLoS One. 2025 Dec 10;20(12):e0338411. doi: 10.1371/journal.pone.0338411 (PMC12694804; doi:10.1371/journal.pone.0338411)

Uncropped agarose gel images used in Figure 14

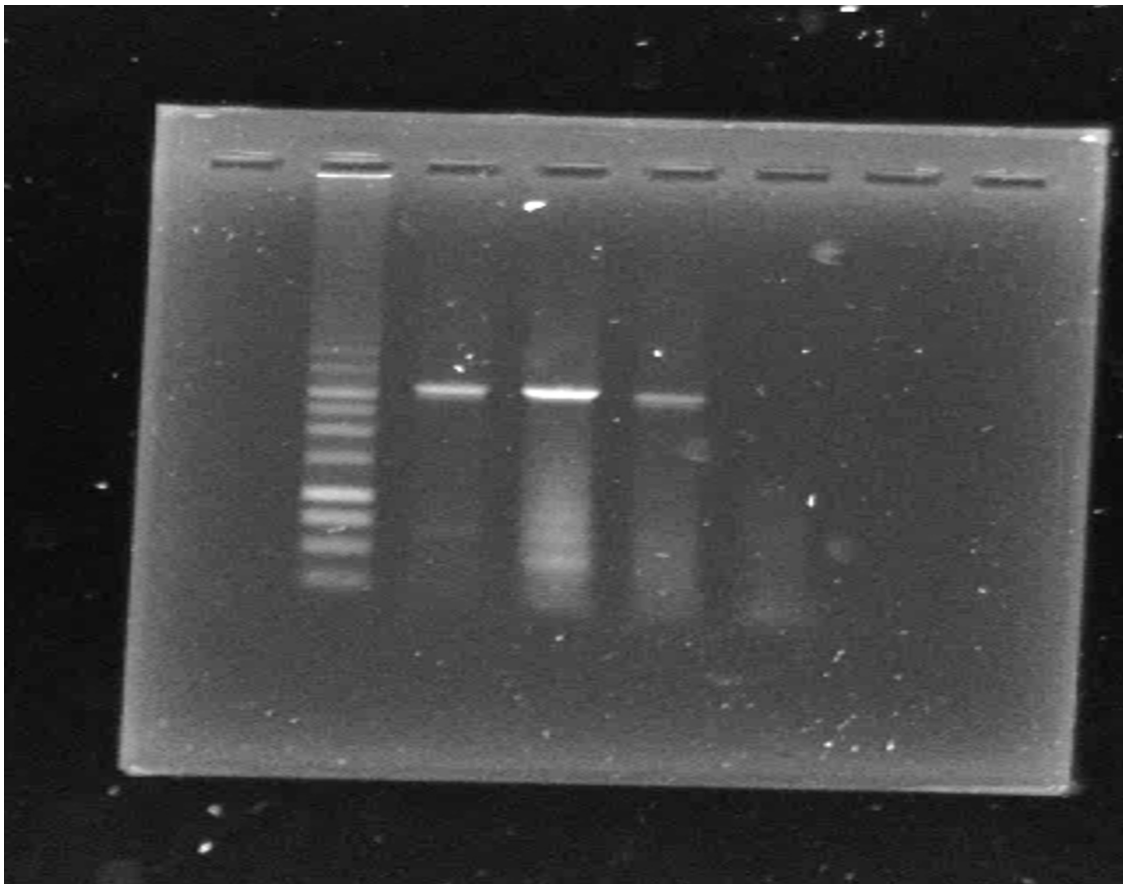

PfIPMK1

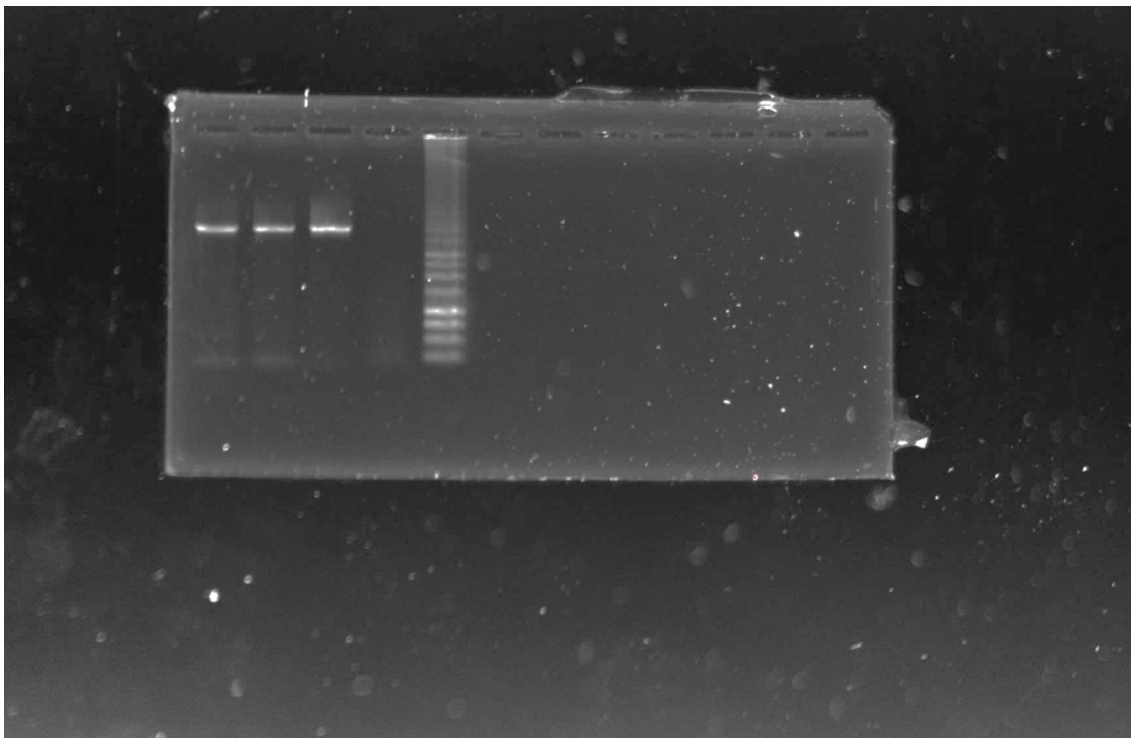

PfVIP

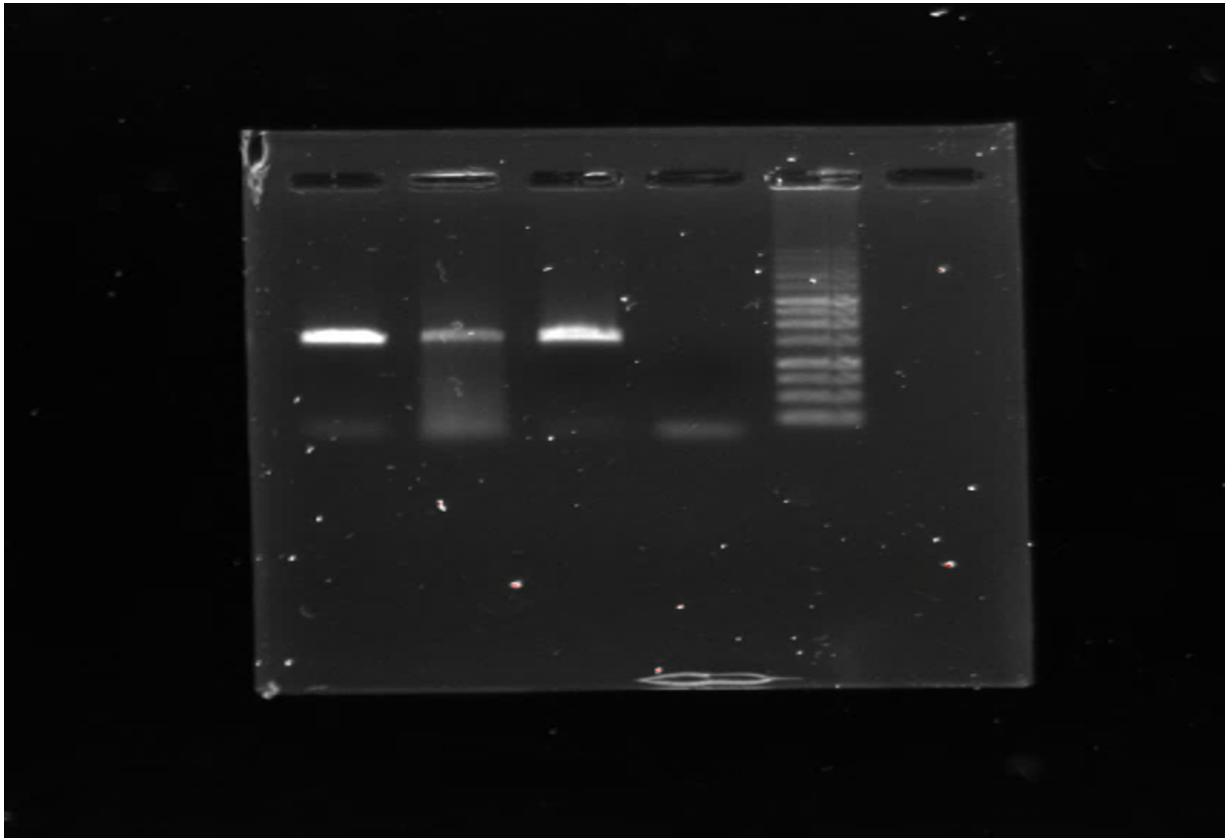

PfIPK2

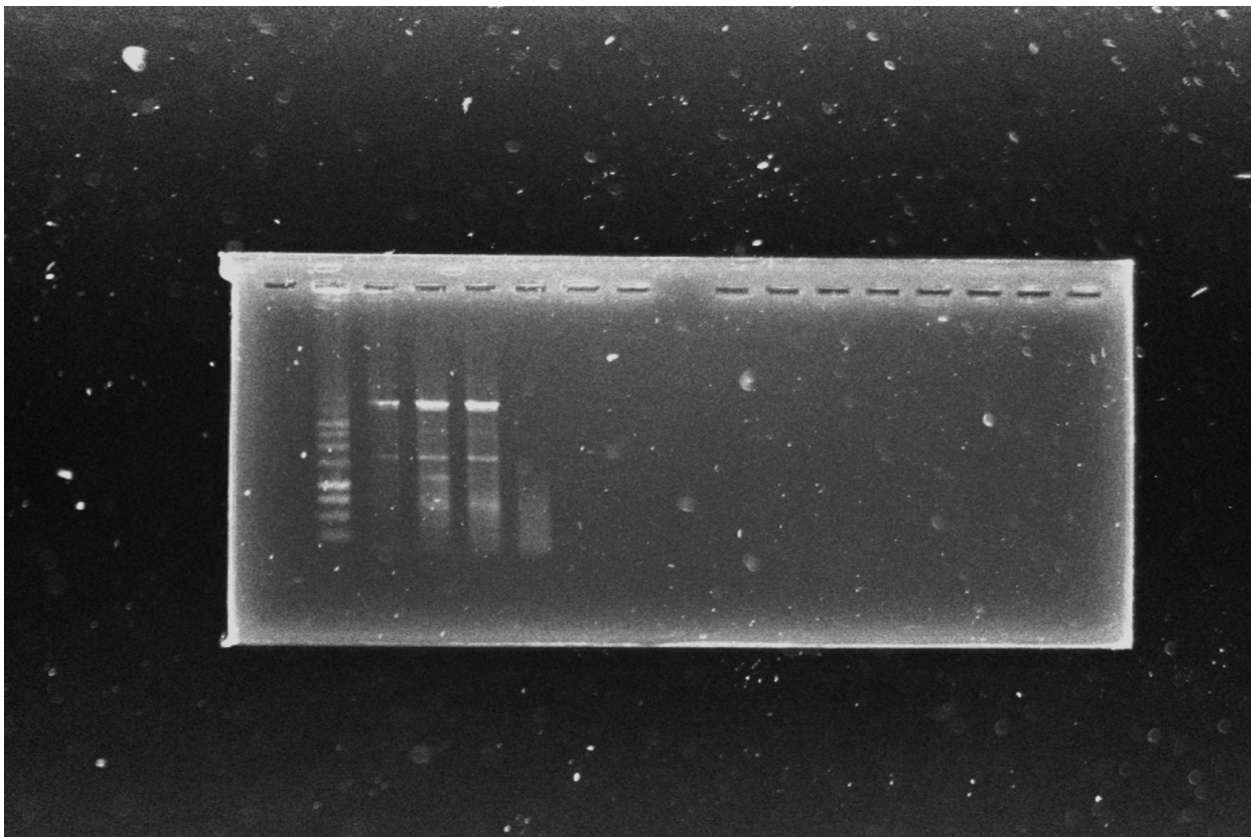

PfIP6K

Supplement: S1 File — Sequences from P. falciparum (Q8I3W0), P. malariae (A0A1D3RII6), P. vivax (A0A564ZW73), P. ovale (A0A1D3U8P3) and P. knowlesi (B3L6B8) were aligned with Clustal Omega and viewed in Jalview. The sequences in the grey frame highlight the identified signature motifs QxxxDxKxG, SLL and IDF. Invariant residues are shaded blue. S1 Table. List of IPK proteins used for structural analysis and their annotations. S2 Fig. Modelling and structure assessment of P. falciparum IPMK1. (A & C) Local distance difference test (LDDT) score per position predicted for the five P. falciparum IPMK1_kinase domain models generated by Colabfold. The model predictions had high- and low-confidence regions, and the low-confidence areas (red, blue and orange frames) were intrinsically disordered regions. (B) PAE plot of the best model predicted, which was refined and analysed on ProSA-Web (E) and SAVES (D), showing that 94.5% was Ramachandran favoured. S3 Fig. 3D modelling of the PfIPMK kinase domain by SWISS-MODEL. (A) Template information. (B) Model assessment of the predicted P. falciparum IPMK2 domain showing a Ramachandra plot of the initial model structure and that of the refined model, which has 94.4% of its residues in the favoured regions (D). (C) ProSA-web output of the quality assessment of the refined model. S2 Table. Table of druggable pockets predicted by PockDrug for P. falciparum IPMK2 and their parameters. S3 Table. Summary of the predicted subcellular locations of the putative P. falciparum IPKs. S1 Sequence. Predicted amino acid sequence of P. falciparum 3D7 IPMK1. S4 Table. Peptides mapped to enzymes in the P. falciparum IPP. S1 document. Uncropped images. (ZIP) [file pone.0338411.s001.zip › Supp_Information/S1 Document.pdf]
