# Supplementary material for: Unveiling the Plasmodium inositol (pyro)phosphate pathway: Highlighting inositol polyphosphate multikinase as a novel therapeutic target for malaria
Source: PLoS One. 2025 Dec 10;20(12):e0338411. doi: 10.1371/journal.pone.0338411 (PMC12694804; doi:10.1371/journal.pone.0338411)
Supplement: Attachment — Submitted filename: Review_PLOS_ONE_05012025.pdf [file pone.0338411.s002.pdf]

In the manuscript titled 'Unveiling the Plasmodium inositol (pyro)phosphate pathway: highlighting inositol polyphosphate multikinase as a novel therapeutic target for malaria', the authors used computational approaches to annotate IPKs, with a special focus on IPMK, within the context of the inositol phosphate pathway. They present IPMK as a promising therapeutic target for P. falciparum malaria. The manuscript includes a comparative analysis of four selected PfIPKs against their human counterparts and homologues from other organisms. While the study adds to understand the molecular landscape of the IPP pathway, there are many major concerns and points need attention and clarification. While the authors have made commendable efforts in analyzing the tertiary structures of the proteins, there remains considerable scope for improving the interpretation and integration of these structural insights into the overall findings.

Most of the analyses presented in the study are computational. Although the authors amplified and sequenced the IPK genes, this adds little new information apart from confirming a previously known change in the IPMK1 motif. In several instances, the conclusions drawn either lack strong supporting evidence or restate already known facts. Therefore, the authors should carefully revise their conclusions and ensure that each statement is well-supported and adds value to the study.

The authors state that **'the presence of their genes was confirmed via conventional PCR and sequencing'** and report amplifying and sequencing the gene from two laboratory strains (Pf3D7 and PfDD2) and one clinical isolate. However, if the primers were designed using sequences from PlasmoDB or NCBI — which contains annotated and validated gene models from the P. falciparum reference genome — confirming the mere presence of these genes seems unnecessary, as their existence in the genome is already well established. Such an approach would only be justified if the study involved highly divergent isolates or aimed to detect strain-specific variation. In this case, the authors appear to be validating a known mutation already reported in the reference genome. While confirming this mutation in the clinical isolate is reasonable, re-sequencing 3D7 and DD2 for the same appears redundant. The authors should revise the statement to clarify that the objective was to validate the mutation, not the gene's presence — or provide a clear rationale for performing this step or for the wording used.

In the section **'Phylogenetic analysis and comparative genomics'**, the authors compare the kinase protein of P. falciparum with its human homologues using both BLASTp and pairwise alignment. However, it is unclear whether the full-length protein or only the kinase domain was used in these analyses. If only the domain was considered, the authors should explain the notable differences in percentage similarity observed between the two methods, particularly in the case of the VIP1 gene. While it is understood that BLASTp performs local alignment and pairwise alignment can be either local or global, the discrepancy in similarity results is difficult to reconcile without clarification. These two paragraphs require revision to ensure consistency and to support a unified conclusion.

Line: 353-355 The authors first state that the QxxxDxKxG motif is specific to P. falciparum, but in the following paragraph, they report its presence in all plasmodial IPMK1 sequences. This appears

contradictory and should be clarified. Moreover, since most plasmodial IPMK1 proteins contain the QxxxDxKxG motif, as opposed to the more commonly reported PxxxDxKxG motif in other IPKs, it may be more accurate to describe this motif as a distinguishing feature of plasmodial IPMK1. This pattern is also supported by other parts of the manuscript and by previously cited studies. The authors are encouraged to reconsider how they frame and interpret this finding.

Line: 366-370: It is important to note that the cited study (ref no. 68) compared PkIPK1, PkIPK2, and HsIPMK, but not PkIPMK specifically. Although the protein XP_002259666.1 (referred to by the authors as PkIPMK1) is mentioned in the cited paper, the study does not provide further discussion on the PDKG motif. Therefore, the authors should revise the statement carefully to accurately reflect the content and limitations of the cited reference.

“**When P. falciparum IP6K is compared with H. sapiens IPK enzymes, it is more closely related to H. sapiens IP6Ks than to H. sapiens IPMK, as indicated by the percentage sequence identity above.”** Isn’t it obvious that P. falciparum IP6K will be more similar to human IP6Ks than to IPMK. Unless this comparison addresses a specific ambiguity or unexpected result, it may not be necessary. The authors could clarify the motivation behind highlighting this point.

The paragraph (lines 463–467) refers to Figure 6 to demonstrate the syntenic nature of IPMK1; however, the figure appears to depict a phylogenetic tree of the VIP1 gene instead. The authors should clarify this discrepancy and ensure that the correct figure is cited.

“**P. falciparum IPMK1 is evolutionarily older and has a mutation that probably impairs its function, thus eliminating functional redundancy.”** How did the authors conclude that PfIPMK1 is evolutionarily older? In Figure 5, what does the branch lengths represent? If it is substitutions per site, which reflect evolutionary change rather than time unless a molecular clock model is used. Additionally, in an earlier section, the authors have shown that PfIPMK1 has distinct motifs compared to homologs in other organisms—differences more likely to result from divergence over time. This seems contradictory to the claim of it being 'older.' Could the authors clarify the basis for this statement and explain how they infer evolutionary age in the absence of a time-calibrated phylogeny?

It would be interesting if authors correlate the active pocket residues and location of the motif spatially and the region which contribute to the lower RMSD value, which might have further strengthen the hypothesis.
