# Supplementary material for: Unveiling the Plasmodium inositol (pyro)phosphate pathway: Highlighting inositol polyphosphate multikinase as a novel therapeutic target for malaria
Source: PLoS One. 2025 Dec 10;20(12):e0338411. doi: 10.1371/journal.pone.0338411 (PMC12694804; doi:10.1371/journal.pone.0338411)
Supplement: Attachment — Submitted filename: Abigail Obuobi et al.docx [file pone.0338411.s003.docx]

Abigail Obuobi et al. in their manuscript has described the importance of inositol phosphate multi-kinases (IPMK) as attractive drug targets against malaria. The authors have talked about the durability of IPMK and the selectivity the drugs will have due to their differences in structure from the human counterpart.

The plasmodium IPMK has a novel variation as described in result “Identification of conserved motifs and residues” which could potentially alter the structure of the protein.

Structural analysis also suggests these proteins are different from the human counterpart making them an attractive target in drug discovery against malaria.

The work however lacks conviction due to the lack of *in-vitro* work supporting the *in-silico* part which is only limited genomic DNA PCR to confirm the presence of the IPMK in the genome of plasmodium.

More *in-vitro* work needs to be done to check the expression levels and pattern of these proteins. (Western blot if antibodies are available or human antibodies can detect these proteins)

Real time PCR to check or the transcription of these genes and role in life cycle stages etc.

Screen compound libraries like the inositol phosphate libraries on plasmodium blood culture and human cell lines to look for inhibition and selectivity of already known inhibitors of these proteins.
